# Discovery and management of arteriovenous malformation with jejunal diverticulum: elusive gastrointestinal bleed

**DOI:** 10.1093/jscr/rjag733

**Published:** 2026-08-23

**Authors:** Annetta Panayides, Maeve E Tremis, Charles Lu, Glenn S Parker

**Affiliations:** School of Medicine, St. George’s University, University Centre, True Blue, St. George's, Grenada, West Indies; School of Medicine, St. George’s University, University Centre, True Blue, St. George's, Grenada, West Indies; Department of Surgery, Hackensack Meridian Health, Jersey Shore University Medical Center, Neptune, NJ 07756, United States; Department of Surgery, Hackensack Meridian Health, Jersey Shore University Medical Center, Neptune, NJ 07756, United States

**Keywords:** angiodysplasia, obscure GI bleeds, jejunal diverticuli

## Abstract

Gastrointestinal (GI) bleeds can pose significant life-threatening situations if not identified promptly, progressing to hemorrhagic shock. We discuss the case of a 60-year-old man presenting with an elusive GI bleed. Esophagogastroduodenoscopy, colonoscopy, Meckel’s scan, and computed tomographic angiography were performed, which yielded no active bleed despite active hemorrhage. As a result, double-balloon computed tomography enterography was performed, demonstrating jejunal diverticula with angiodysplasia and arteriovenous malformations (AVMs), suspected to be the source of the elusive GI bleed. Clips were attempted at the sites of visible AVMs, but were unsuccessful. The diverticula were tattooed with a plan for an exploratory laparotomy for definitive treatment. The patient was ultimately discharged with no signs of active bleeding and an uncomplicated recovery. This case highlights the multifactorial management of a massive GI bleed with no identifiable source of bleeding upon admission, progressing into hemorrhagic shock.

## Introduction

Obscure gastrointestinal (GI) bleeding remains a significant diagnostic challenge, accounting for ~5%–10% of GI hemorrhages [1, 2]. Vascular lesions originating in the small bowel are one of the most common causes, particularly angiodysplasias, including arteriovenous malformations (AVMs) and Dieulafoy lesions. Angiodysplasias are dilated, tortuous, thin-walled vessels within the mucosal and submucosal layers of the bowel that are susceptible to intermittent bleeding [3]. Structural abnormalities, including jejunal diverticula, can contribute to bleeding or complicate the localization of the true source. The small size, intermittent bleeding, multifocal distribution, and difficult anatomic location of these abnormalities make detection challenging, particularly when hemorrhage is inactive during evaluation [1, 3, 4].

Obscure GI bleeding refers to persistent or recurrent bleeding after nondiagnostic esophagogastroduodenoscopy and colonoscopy, with or without capsule endoscopy [1, 2]. If the bleeding source remains elusive with intermittent hemorrhage or negative initial studies, computed tomography enterography (CTE) can identify mural, vascular, and structural abnormalities that are not readily detected on endoscopy or standard angiographic studies [4, 5].

We present the case of recurrent, life-threatening gastrointestinal bleeding in which an extensive diagnostic evaluation failed to localize the source of hemorrhage. Ultimately, CTE identified the bleeding source as a rare occurrence of jejunal diverticula with AVMs. This case underscores the limitations of conventional diagnostic modalities in obscure GI bleeding and highlights the clinical utility of CTE in detecting small-bowel vascular lesions.

## Case presentation

A 60-year-old man presented to the emergency department with generalized weakness, lightheadedness, and melena. He had been hospitalized previously for rectal bleeding and underwent esophagogastroduodenoscopy, colonoscopy, and capsule enteroscopy, which were all unrevealing. He was subsequently discharged home.

On repeat presentation, the patient was hypotensive requiring vasopressor support with a hemoglobin level of 5.8 g/dl. He was admitted to the intensive care unit for hemodynamic stabilization and transfusion of multiple packed red blood cells for hemorrhagic shock.

His past medical history was notable for gastroesophageal reflux disease, Lyme disease, and syncope. During the current admission, further evaluation included a technetium-99m pertechnetate (Meckel) scan and computed tomography angiography (CTA), both failing to identify a bleeding source.

**Figure 1:**
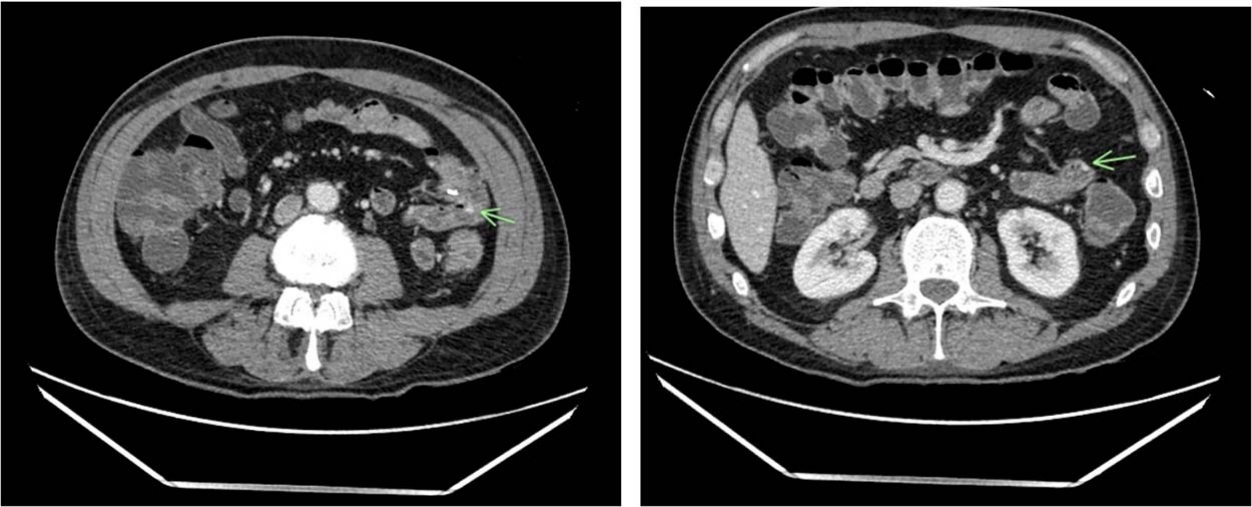
CTE axial view demonstrating AVMs on the venous phase proximal to a previous clip (arrows).

**Figure 2:**
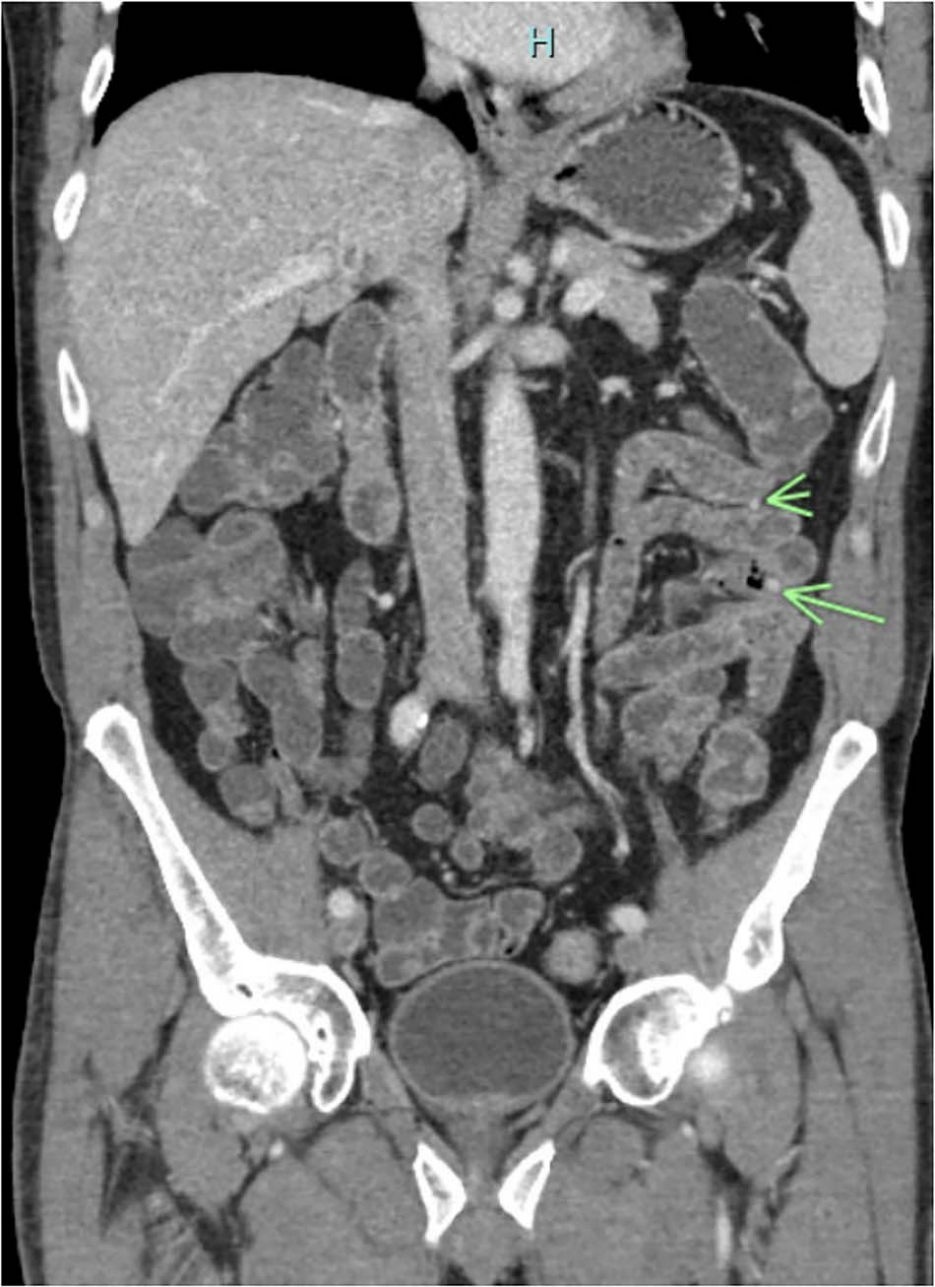
CTE coronal view demonstrating multiple AVMs on the jejunum (arrows).

Given ongoing concern for gastrointestinal hemorrhage, a repeat colonoscopy demonstrated black effluent within the terminal ileum, raising suspicion for small-bowel bleeding. Despite supportive management, the patient’s hemoglobin continued to progressively decline, promoting CTE. The bleeding source was localized with multiple small-bowel AVMs in the venous phase near a prior colonoscopy clip (Figs 1 and 2).

**Figure 3:**
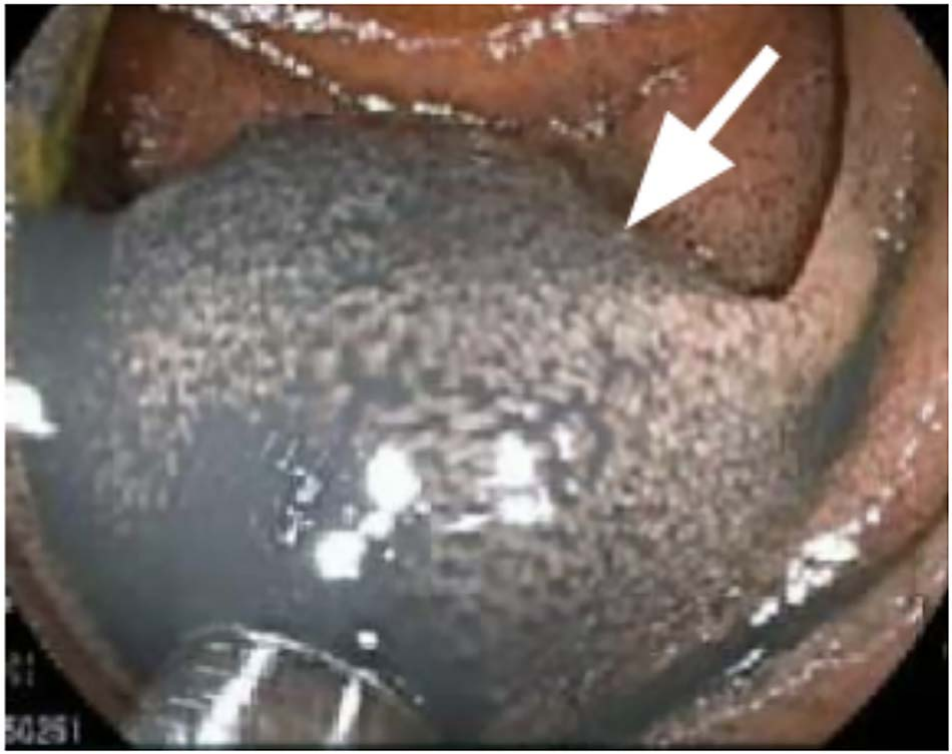
Double–balloon–assisted enteroscopy of the mid-ileum demonstrating the tattoo of the bleeding region (arrow).

**Figure 4:**
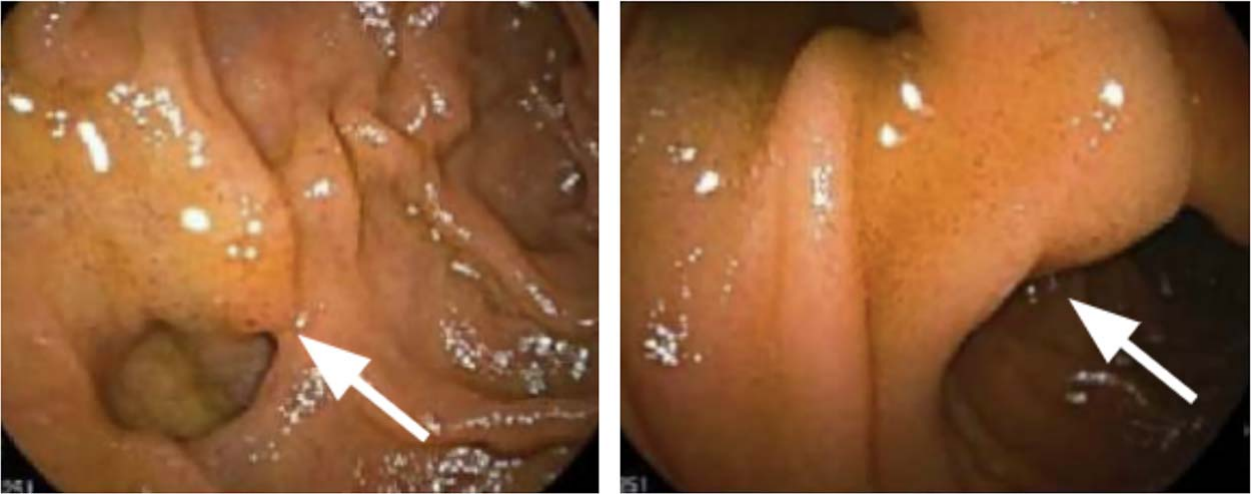
Double–balloon–assisted enteroscopy of the jejunum demonstrating diverticula (arrows).

The patient then underwent double-balloon–assisted enteroscopy (DBE), and the identified bleeding regions were tattooed (Fig. 3). Endoscopic evaluation revealed ~45 cm of small bowel involvement with multiple diverticula within the jejunum (Fig. 4).

At this point, surgical intervention was indicated. The patient underwent a proximal jejunal resection, removing ~30 cm of tattooed jejunal diverticula and an additional 15 cm of jejunum, followed by primary anastomosis. Four specimens were submitted for histopathologic evaluation; the proximal jejunum demonstrated multiple diverticula and underlying prominent blood vessels, the distal jejunum demonstrated moderate congestion with prominent submucosal blood vessels confirming angiodysplasia, and the small bowel proximal staple line and distal jejunal staple line demonstrated moderate congestion.

The patient’s postoperative course was uncomplicated, and he was discharged home in stable condition.

## Discussion

Jejunal diverticula are uncommon, with a prevalence of 0.3%–5.0%, and co-existing with AVMs is even rarer, typically manifesting as gastrointestinal hemorrhage [6]. The novelty of this case lies in the rare coexistence of jejunal diverticula and AVMs causing obscure bleeding despite guideline-directed investigations. Ultimately, CTE and DBE localized occurrence of jejunal diverticula with AVMs. This case illustrates the limitations of standard algorithms in diagnosing elusive GI bleeds.

Capsule endoscopy and colonoscopy are first-line investigations for suspected small-bowel bleeding, successfully identifying bleeding sources in 45%–90% of cases [7, 8]. In this case, colonoscopy and endoscopy were unrevealing, highlighting the diagnostic challenge. Furthermore, capsule endoscopy may yield limited information in cases of rapid bleeding, intramural pathology, or lesions extending beyond the mucosal surface [2].

Applying the algorithmic approach proposed by Petersile et al. (2019) to this case revealed several limitations [7]. Nasogastric lavage was omitted due to prior negative esophagogastroduodenoscopy, but both gastroenterology and surgical consultations were obtained as recommended. CTA was indicated, given the patient’s hemodynamic instability with normal renal function (creatinine <1.7 mg/dl). Active bleeding at rates ≥0.5 ml/min can be detected using CTA, with reported sensitivities of 86% and specificities of 95%. In addition, CTA has been associated with reduced blood transfusion requirements (mean 1.3 vs 2.2 units, *P* = .01) [7–9]. In our case, CTA yielded a false-negative, likely due to transient cessation of bleeding or bleeding below the detection threshold at the time of imaging [10].

The algorithm does not provide explicit guidance if CTA is negative, underscoring the rarity and complexity of this case. Given a recent negative colonoscopy within 3 months, a Meckel scan was obtained for suspected small-bowel bleeding. Following a negative result, tagged red blood cells scanning for slower bleeding (≥0.1 ml/min) could have been considered, but was not performed [7, 8].

Ultimately, CTE localized the bleeding source and identified jejunal diverticula with AVMs, demonstrating its efficacy in detecting mural, vascular, and extraluminal pathology not visualized endoscopically [11]. Subsequently, DBE was employed for definitive diagnosis, demonstrating a sensitivity of 84% and specificity of 92% for detecting stable-to-mild-to-moderate active GI bleeds [12]. This case emphasizes the limitations of the algorithmic approach in identifying rare, complex pathologies, emphasizing the need for advanced imaging in obscure GI bleeds.

Despite multiple interventions, GI bleeding can require operative management. A laparoscopic approach was not considered due to the patient’s hemodynamic instability secondary to hemorrhagic shock. Ultimately, this patient required an exploratory laparotomy with a small bowel resection as definitive treatment.

This case highlights the severity of GI bleeding caused by AVMs coexisting with jejunal diverticula. Early detection of these pathologies is crucial to prevent adverse outcomes and treatment delays.

## Conclusion

Jejunal diverticula with AMVs can present with GI bleeding progressing to hemorrhagic shock requiring immediate interventions. In this patient, CTE and DBE ultimately identified the source of the bleed after a negative diagnostic evaluation, resulting in a jejunal resection. Early recognition of GI bleeding is vital for early intervention and to prevent delays in care.
